# Genetic structure of *Bithynia* snails and its implications for targeted control of opisthorchiasis in the Lower Mekong Subregion

**DOI:** 10.1186/s41182-025-00774-w

**Published:** 2025-08-15

**Authors:** Nathkapach Kaewpitoon Rattanapitoon, Chutharat Thanchonnang, Schawanya Kaewpitoon Rattanapitoon

**Affiliations:** Parasitic Disease Research, FMC Medical Center of Thailand, Nakhon Ratchasima, 30000 Thailand

**Keywords:** Bithynia snails, Genetic structure, *Opisthorchis viverrini*, Targeted control, Lower Mekong Subregion

## Abstract

Recent findings by Bunchom et al. reveal distinct mitochondrial lineages of *Bithynia siamensis goniomphalos* in Champasak, Laos, with important implications for *Opisthorchis viverrini* transmission. The identification of lineage II, with high haplotype diversity and habitat-specific distribution, underscores the role of snail genetics in shaping local disease dynamics. Drawing parallels from Thailand’s successful One Health intervention, this correspondence advocates for integrating lineage-informed mapping and surveillance into regional control programs. Broader genetic monitoring of Bithynia snails across the Lower Mekong Subregion may enhance the precision and effectiveness of liver fluke elimination strategies.

Dear Editor,

We read with great interest the recent article by Bunchom et al. [1], which provides compelling evidence of significant genetic structuring in *Bithynia siamensis goniomphalos* populations from Champasak Province, Laos. Their discovery of distinct mitochondrial lineages (II and III) and the absence of lineage I in the study area suggest strong geographic partitioning and the presence of natural gene flow barriers, such as the Li Phi (Somphamit) waterfall. This finding carries profound implications for the design of sustainable liver fluke control programs.

The importance of local snail genetics in shaping transmission patterns of *Opisthorchis viverrini* is increasingly recognized. The study highlights that lineage II, newly reported in Laos, displays high haplotype diversity and genetic isolation from Cambodian populations. Given prior research indicating variable susceptibility of *Bithynia* lineages to *O. viverrini* infection [2–4], these findings suggest that lineage composition may influence local parasite transmission intensity. Notably, lineage II is associated with temporary habitats, potentially promoting high intra-population gene flow and amplifying local transmission.

The impact of lineage-informed control strategies is already evident in Thailand. For instance, the One Health program in Udon Thani Province integrated ecological, parasitological, and behavioral data to reduce human opisthorchiasis from 14.1% to below 1% over 6 years [5]. A key factor in this success was the recognition of high-risk ecological zones and targeted snail control. Extending this logic, fine-scale mapping of *Bithynia* genetic lineages could further sharpen intervention strategies in transboundary hotspots like southern Laos.

The study by Bunchom et al. also affirms the need for wider genetic surveillance of *Bithynia* spp., especially in understudied regions, such as Myanmar and Vietnam. The absence of *B. funiculata* and *B. s. siamensis* in their study area supports the ecological specificity of *B. s. goniomphalos* and underlines the importance of species-level confirmation via molecular tools.

We commend the authors for generating novel baseline data in a high-burden setting. Their work paves the way for lineage-based surveillance and intervention models. We urge the research and public health communities to integrate genetic data of intermediate hosts into regional opisthorchiasis control efforts—doing so may prove to be the missing piece for breaking transmission in persistent endemic zones.

## Data Availability

No data sets were generated or analyzed during the current study.
